# Establishing trauma databases in underserved regions: a call to action for advancing data-driven surgical quality improvement

**DOI:** 10.1097/JS9.0000000000002881

**Published:** 2024-06-30

**Authors:** Cherinet D. Osebo, Lilly Groszman, Alexander Laskaris, Ying Chen, Respicious L. Boniface, Victoria J. Munthali, Ibrahima Konate, Nathalie Boulanger, Yidnekachew D Gone, Evan Wong, Dan L. Deckelbaum, Jeremy R. Grushka

**Affiliations:** a Emergency Surgery and Obstetrics Units, Hargele Hospital, Hargele, Ethiopia; b Department of Medicine, Primary Care and Population Health Division, Stanford University, Stanford, California, USA; c Department of Surgery, Trauma and General Surgery Division, Centre for Global Surgery, Montreal General Hospital, McGill University, Montreal, Canada; d Department of Anesthesiology, Muhimbili Orthopedics Institute, Dar es Salaam, Tanzania; e Department of Orthopedics, Muhimbili Orthopedics Institute, Dar es Salaam, Tanzania; f Department of Surgery, Centre Hospitalier Régional de Saint Louis, Gaston Berger University, Senegal; g Department of Emergency and Critical Care, Nigist Elleni Mohammed Memorial Hospital, Wachemo University, Hosaena, Ethiopia; h Department of Surgery, Ungava Tulattavik Health Centre, Kuujjuaq, Northern Quebec, Canada

## Introduction

Millions of preventable trauma deaths occur annually due to limited surgical access, exacerbated by gaps in data-sharing infrastructure, hindering evidence-based policymaking in low-resource settings[1]. Unstandardized surgical data impede care optimization and policy effectiveness, necessitating comprehensive data collection to identify care gaps and improve outcomes—especially in regions where infrastructure and workforce capacity remain severely constrained.

To address these gaps, McGill University’s Centre for Global Surgery (CGS) developed a low-cost, browser-based, offline-capable trauma data platform in 2022. Built through international academic partnerships, this system was specifically designed for use in emergency care settings across underserved regions. It enables real-time, prospective trauma data capture at the point of care while functioning without continuous internet connectivity. This feature, along with built-in validation checks to flag incomplete records and user-friendly dashboards, supports accurate and timely clinical decision-making, identifies injury trends, informs care delivery, and strengthens trauma system responsiveness.

**Figure 1.**
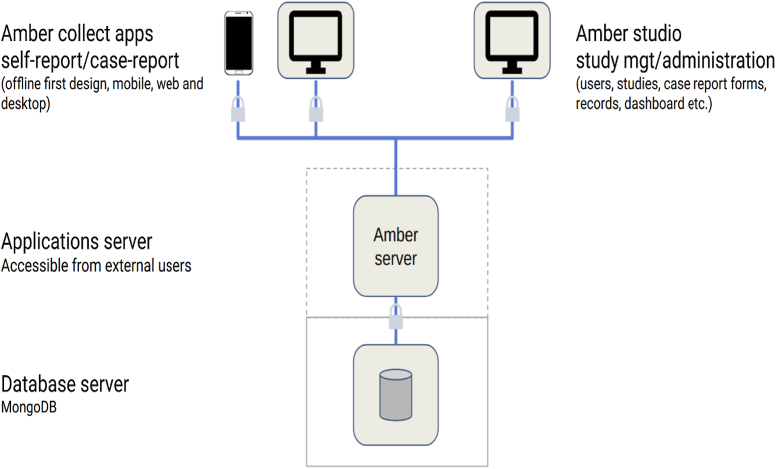
Web-based trauma registry platform’s flow diagram[3].

In compliance with the TITAN Guidelines 2025^2^, which promote transparent and ethical reporting of digital tools in surgical settings, our deployment emphasizes accessibility, equity, and integration with existing workflows. The platform, Amber Collect (cglobalsurgery.ca), consists of three core modules (Fig. 1)^3^: Amber Collect (for point-of-care data entry), Amber Studio (for data review and management), and Amber Server (for secure cloud-based storage). Data can be entered on any device with a browser, stored offline, and automatically synchronized when network access is restored.

**HIGHLIGHTS**

- Offline web-based trauma databases improve data accuracy, decision-making, and surgical outcomes in underserved regions.
- Cross-country studies (Tanzania, Senegal, Ethiopia, and Nunavik [Canada]) demonstrate the impact of structured trauma data on care improvements.
- Data completeness increased from 50 to 67% (paper-based) to 100%, optimizing patient management.
- Automated analysis reduced reporting time from days to hours, enabling real-time decision-making.
- In Tanzania, database insights led to improved triaging, reducing arrival-to-care times by 55% and trauma mortality by 42%.
- Expansion to operating theaters enhanced surgical resource allocation and workflow.
- Local stakeholder engagement, workforce training, and infrastructure investment are key for sustainability.
- Nunavik’s case highlights challenges in underserved high-income regions, emphasizing the need for targeted interventions.
- This paper calls for urgent action to scale digital trauma databases in other underserved regions to improve surgical care and inform policy.

Unlike global health data platforms like WHO’s district health information software 2 (DHIS2), which mainly focus on aggregated public health metrics, our system captures detailed clinical data—including patient demographics, injury mechanisms, severity scores, procedures, and outcomes. DHIS2 primarily serves as an aggregate-level tool for national health reporting and is not designed for point-of-care clinical decision-making. However, our trauma database addresses a unique gap: individual-level, real-time data collection at the bedside, optimized for trauma workflows, low-connectivity environments, and clinical feedback loops. Furthermore, the database was co-designed with frontline providers to ensure immediate clinical and policy utility in resource-limited emergency settings, where rapid decision-making and data integrity are crucial. Real-time use enhances both clinical care and operational oversight, providing actionable insights that are not possible with retrospective paper-based methods.

To our knowledge, this is one of the first multinational implementations of a real-time, offline-capable trauma registry tailored to both low- and high-income underserved contexts. This paper presents the implementation experience from four diverse settings—Tanzania, Senegal, Ethiopia (low-income, sub-Saharan Africa), and Nunavik (high-income but underserved Indigenous region in Canada)—highlighting early feasibility, lessons learned, and challenges in adapting trauma data systems to variable resource environments. In doing so, we address critical gaps in the literature and provide new evidence on the utility of digital trauma databases in strengthening care quality and informing policy in areas where data-driven trauma system planning is urgently needed.

## Methods

**Figure 2.**
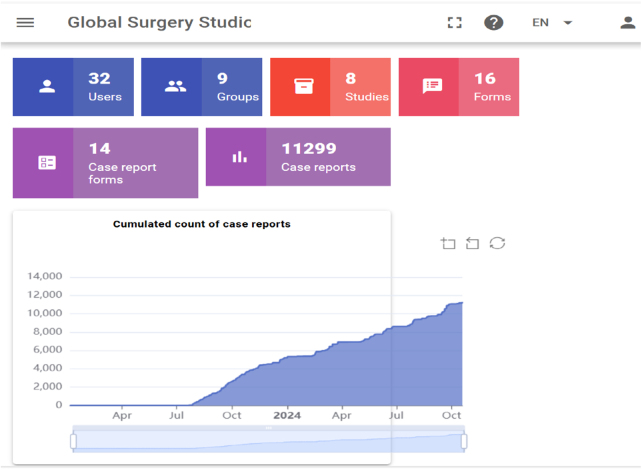
Displays web-based database screenshots capturing cumulative data across settings.

The trauma database was introduced through academic partnerships coordinated by McGill University’s CGS. This paper presents descriptive implementation reports from four underserved regions—Tanzania, Senegal, Nunavik, and Ethiopia—selected based on their unmet trauma data needs. Each site underwent a pre-implementation assessment, including infrastructure audits, clinical workflow mapping, and interviews with clinicians, administrators, data managers, and IT teams to evaluate feasibility, usability, and sustainability. Implementation began in Tanzania in July 2023 with prospective data collection at a tertiary hospital’s emergency unit, serving as a pilot for subsequent rollouts (Fig. 2). Senegal and Nunavik joined in late 2024, and Ethiopia has finalized stakeholder engagement and infrastructure planning to roll out to implementations. All trauma patients presenting to the emergency departments of participating sites were included, with the only exclusion being non-trauma diagnoses.

The platform was offered at no cost and customized to each site’s capacity. It was chosen for its compatibility with low-resource environments, requiring only a browser and allowing offline data entry with automatic synchronization upon internet reconnection. Implementation support included browser-compatible tablets with portable internet access and local troubleshooting guides. Both in-person and virtual training sessions were carried out with clinical, administrative, and IT teams. Workflow integration allowed for point-of-care data entry by trained personnel using bookmarked devices after initial resuscitation to minimize clinical disruption. Data were validated and finalized post-stabilization through the inclusion of 24-hour dispositions and 2-week outcomes. Follow-up past 2 weeks was not feasible due to system and staffing limitations. Ongoing communication between CGS and local teams fosters system sustainability, troubleshooting, and iterative quality improvement. The study complies with the TITAN 2025 Guidelines[2], which promote transparent reporting of digital tools in surgical research. No generative artificial intelligence was used in the writing, analysis, or interpretation of this manuscript.

## Results and discussion

### Insights and lessons learned

#### Tanzania: transforming surgical care through data-driven decisions

In Tanzania, where access to surgery and data-sharing is limited, CGS partnerships have significantly improved trauma care. The web-based trauma database, implemented in 2023 at Muhimbili Orthopedic Institute (MOI), standardizes data collection and enhances surgical outcomes through real-time decision-making[3]. The platform increased data completeness from 67% (paper-based) to 100% by flagging incomplete data, enabling surgical teams to identify care gaps and optimize treatment. For instance, 65% of patients received life-saving surgeries within 30 minutes; however, delays of over an hour affected 15% of cases, resulting in a 3% mortality rate among patients with severe head injuries and fractures. This contradicts the trauma protocol’s golden hour, during which 60% of preventable deaths occur within the first hour^4^. MOI’s systemic challenges, including triaging infrastructure issues, workforce shortages, and reliance on non-ambulance transport for 43% of critically injured patients, contribute to delays, leading to higher injury severity (mean severity score 2.26 ± 0.91, *P* <0.027), increased mortality (mean 0.32 ± 0.11, *P* <0.017), and prolonged hospital arrival times (median 420 [240–600] minutes, *P* <0.004). These findings emphasize the importance of structured databases in identifying care gaps, optimizing resources, and improving surgical equity.

After the trauma database’s success, MOI expanded the platform to capture operating theater (OT) data through customizable questionnaires^[5]^. The system achieved 100% data accuracy. The OT utilization report revealed that among elective cases across seven OTs, with 735 calendar days assigned, a 92% completion rate (*P* <0.011) was reached, and 8% cancellations were documented (mean 1.47 ± 0.19, *P* <0.032). Furthermore, 97% of patients were discharged alive, and a 3% perioperative mortality rate was noted (*P* <0.051), highlighting the database’s value in optimizing OT management, resources, workflow, and patient outcomes.

Leveraging robust databases is essential for identifying gaps and guiding targeted interventions. At MOI, the data revealed fragmented triage and care protocols, which prompted the implementation of Trauma and Disaster Team Response training^[6]^. Led by Tanzanian and CGS faculties, the program trained 32 clinicians, including nurses, physicians, surgeons, and residents, enhancing their skills, teamwork, and confidence (99% satisfaction). Six months post-training, structured triaging reduced arrival-to-care times by 55%, with the mean time decreasing from 29 minutes to 13 minutes (± 1.93, *P* <0.003). Mortality also dropped by 42% (*P* <0.024), highlighting the impact of data-driven strategies in improving trauma care and patient outcomes.

While the observed reduction in mortality is promising, we recognize several methodological limitations. The comparison relies on historical pre-implementation data rather than a contemporaneous control group, which introduces potential biases such as confounding and secular trends. Although injury severity scores were collected and showed significant differences, other baseline variables (e.g., comorbidities, prehospital care, or socioeconomic factors) were not consistently available, limiting our ability to fully adjust for confounding. Furthermore, memory bias and selection bias may affect the interpretation of outcomes. As a result, causality cannot be definitively attributed to database implementation alone. Future studies incorporating matched controls or stepped-wedge designs could more rigorously assess the impact. However, despite its successes, challenges remain, including technical issues and, while the built-in system flags incomplete data, human errors exist—for example, entering age as 777 instead of 77 or systolic blood pressure as 1100 mmHg instead of 110 mmHg, among others.

#### Senegal: data-driven approaches to enhancing surgical care

Trauma in Senegal results in poor outcomes, as the absence of national trauma registries hampers trauma epidemiology, training, and the efficiency of care^[7]^. The needs assessment at Centre Hospitalier Régional de Saint Louis revealed that paper-based methods led to incomplete data^[7]^, complicating policymaking and prompting the adoption of the database, which improved data accuracy and decision-making. Six months of pre- and post-implementation analysis of 358 cases indicated that data completeness rose from 50% (paper-based) to 100%, thanks to built-in error detection. Automated analysis shortened data processing time from weeks to under hours, reflecting Tanzania’s experience, where data processing time fell from over 48 hours to under 1 hour, facilitating real-time decision-making^[3]^.

Local stakeholders support the system due to its impact on policymaking, with surgical teams appreciating its role in identifying injury patterns and care gaps. For instance, clinicians noted, “The system allows us to detect trauma case trends, enabling effective surgical resource allocations.” Administrators highlighted its effect on operational transparency, real-time patient tracking, and evidence-based policymaking. However, challenges such as limited digital proficiency, resource shortages, and software glitches necessitate ongoing training and technical improvements. While CGS support is vital, continued local funding guarantees long-term impacts for dedicated clinicians and policymakers, enhancing surgical care.

#### Nunavik: addressing current trauma care challenges

Canada’s Quebec regionalized that trauma systems were implemented in 1993, reducing trauma mortality from 52% to 9% within a decade^[8]^. However, northern Quebec’s Nunavik, an Indigenous region, faces trauma rates 4 times higher and mortality rates 40 times higher than the provincial average^[9]^. Currently, the region lacks trauma registries, and Quebec systems only capture data for patients transferred to tertiary care centers, leaving significant trauma cases unrecorded, which restricts efforts to enhance care^[8,9]^. To address this, the database was introduced at Ungava Tulattavik Health Centre in Kuujjuaq, one of two Nunavik referral centers. A review of 559 trauma cases for the needs assessment highlighted the region’s high trauma burden, emphasizing the necessity for structured data systems.

Early findings from 77 post-implementation cases collected at the pilot phase revealed key injury trends: blunt trauma (24%, 19/77), falls (20%, 15/77), and penetrating injuries (15%, 12/77), with most trauma originating from Kuujjuaq. This time-bound sample was selected to reflect the immediate impact following database deployment. To ensure data integrity, cases were entered in real time by trained staff using automated validation features, minimizing transcription errors. While most patients (74%, 57/77) were discharged the same day, 12% (9/77) were admitted, and 8% (6/77) were referred to a higher facility. The limited trauma case volume may reflect seasonal variation in injury rates.

Key stakeholders noted that while existing paper-based charting took 1–2 months to code, the new system enables real-time data entry and error detection, improving data completeness, access, and reporting time. Despite challenges like administrative burdens and limited trained personnel, it enhances trauma care by mapping injury trends and guiding care planning. Nunavik’s case underscores unique challenges in high-income, underserved regions.

#### Ethiopia: bridging data gaps for improved surgical services

Ethiopia faces significant surgical challenges: low surgical volumes and surgeon density (43 and 0.54 per 100,000, respectively), leaving 80% of rural areas without timely care^[10]^. The lack of reliable databases hinders evidence-based decision-making, as shown by discrepancies in perioperative mortality reporting—1.4% in government reports vs. 3.3% in published studies^[11]^. The national Saving Lives Through Safe Surgery initiative achieved a 25% reduction in surgical complications over 3 years using data-driven strategies, yet its 2025 goals (Table 1)—including establishing structured data-sharing systems—remain partially unmet due to technical and policy-level constraints^[12]^.

**Table 1:** Ethiopian Ministry of Health Targets for the National Surgical Plan 2025

Achieve 100% national POMR tracking system^a^
Reduce delay for elective surgery admission from 51 to 30 days
Conduct 2500 procedures per 100,000 population by the end of 2025
Reduce the perioperative mortality rate to <2%
Achieve 100% tracking of surgical care-related deaths
Reduce the SSI rate to <5%
Increase the cesarean section rate from 4% to 10%
Provide 100% of woredas (districts) access to essential surgical care
Reduce anesthesia adverse events by 50%
Achieve 100% utilization of the SSC
Increase percentage of facilities providing basic surgical services from 44% to 80%
Reduce the number of clients on the waiting list for elective surgical services by 50%
Increase the proportion of health facilities with electricity from 76% to 100%
Increase the proportion of health facilities with an improved water supply from 59% to 90%

^a^ POMR, perioperative mortality rate; SSC, surgical safety checklist; SSI, surgical site infection.

Existing platforms like DHIS2, while valuable for aggregate reporting, are rigid and lack the capacity for real-time clinical decision support, case-level tracking, or feedback loops necessary for frontline care improvements. In response, Wachemo University Hospital is implementing a web-based trauma and surgical database to replace error-prone paper records and complement DHIS2. The system allows for real-time data entry, clinical quality tracking, and care gap identification. Although integration with DHIS2 is ongoing, early implementation has focused on parallel data collection with efforts underway to align core data fields and ensure interoperability. Challenges remain, including limited digital infrastructure, workforce training needs, and a lack of clear data governance frameworks, but the platform offers a promising step toward improved surgical care and policy responsiveness.

#### Addressing challenges

Implementing the web-based trauma database in underserved regions required addressing technical and human-related obstacles. Commonly encountered issues include inconsistent connectivity and occasional software glitches. To mitigate these, the platform was updated to support automatic syncing and offline data entry, with tablets equipped with SIM-based access provided to ensure continuity. Unlike aggregate-focused systems like DHIS2, this platform enables case-level tracking, immediate feedback loops, and real-time clinical decision support, which are essential for surgical care improvement. Comprehensive troubleshooting guides and regular training empowered local teams to address minor issues independently. Measures like random cross-checks identified and corrected common human entry errors (e.g., age recorded as 777 instead of 77), thereby improving data accuracy. The system also supports automated dashboards and visualization tools to track data completeness and clinical trends, facilitating use in local quality improvement meetings. Continuous communication channels between local site liaisons and the CGS team allowed for real-time problem-solving and technical support. Though challenges remain—such as infrastructure limitations and the need for workforce capacity building—this iterative, collaborative approach enhances the system’s reliability, sustainability, and suitability for use in data-poor environments.

## Conclusion

Surgical data is essential for improving patient outcomes through data-driven decision-making. Experiences across diverse settings in this study demonstrate the real-world value of implementing trauma databases to identify care gaps, guide clinical quality improvement, and inform policy. Achieving equitable surgical care in underserved regions requires committed partnerships, strategic system strengthening, and targeted investments. Platforms introduced in these settings show that web-based trauma databases advance error-prone paper-based systems and can complement rigid national systems by enabling real-time entry, case-level tracking, and feedback loops—functions critical to frontline care but lacking in current aggregate-reporting platforms. While these efforts are still in early stages, they offer a pragmatic and scalable model for other settings facing similar data infrastructure gaps. To ensure sustainability and measurable impact, we call for the following targeted actions:

- **Policy Integration**: Embed trauma data systems into national health frameworks and explore pilot policies to test integration models and interoperability with existing tools.
- **Capacity Building**: Develop and fund training programs to build local expertise in digital data collection, maintenance, and analytics.
- **Infrastructure Investment**: Support the procurement and maintenance of reliable digital infrastructure, with a focus on rural deployment and addressing internet access challenges.
- **Stakeholder Collaboration**: Formalize multisector partnerships among clinicians, policymakers, and tech developers to tailor systems to clinical realities and policy needs.
- **Monitoring and Improvement**: Design continuous quality improvement loops and consider research frameworks with control groups or cost-effectiveness analysis to validate impact.
- **Academic Partnerships**: Collaborate with global academic and public health institutions to co-develop tools, share insights, and support the scalable implementation of these initiatives.

These recommendations are aligned with the strategic pillars outlined in national surgical planning efforts and global surgery initiatives, offering a practical roadmap for digital system integration in underserved settings. By investing in sustainable, interoperable digital data systems and applying real-world lessons, countries can drive measurable improvements in trauma care and move closer to the global goal of *halving preventable deaths* by 2050^[13]^ .

## Data Availability

Available upon reasonable request.
